# Evolution of CRISPR/Cas Systems for Precise Genome Editing

**DOI:** 10.3390/ijms241814233

**Published:** 2023-09-18

**Authors:** Magdalena Hryhorowicz, Daniel Lipiński, Joanna Zeyland

**Affiliations:** Department of Biochemistry and Biotechnology, Poznan University of Life Sciences, Dojazd 11, 60-632 Poznań, Poland; daniel.lipinski@up.poznan.pl (D.L.); joanna.zeyland@up.poznan.pl (J.Z.)

**Keywords:** CRISPR/Cas9 system, CRISPR classification, Cas9 nuclease, Cas12a nuclease, Cas13a nuclease, genome editing, base editors, prime editors

## Abstract

The bacteria-derived CRISPR/Cas (an acronym for regularly interspaced short palindromic repeats/CRISPR-associated protein) system is currently the most widely used, versatile, and convenient tool for genome engineering. CRISPR/Cas-based technologies have been applied to disease modeling, gene therapies, transcriptional modulation, and diagnostics. Nevertheless, some challenges remain, such as the risk of immunological reactions or off-target effects. To overcome these problems, many new methods and CRISPR/Cas-based tools have been developed. In this review, we describe the current classification of CRISPR systems and new precise genome-editing technologies, summarize the latest applications of this technique in several fields of research, and, finally, discuss CRISPR/Cas system limitations, ethical issues, and challenges.

## 1. Introduction

The CRISPR/Cas (an acronym for regularly interspaced short palindromic repeats/CRISPR-associated protein) system revolutionized genetic engineering research by significantly improving the efficiency and accuracy of genome editing. The CRISPR/Cas system was initially discovered in bacteria and archaea as an adaptive prokaryotic immune system to defend against invasive nucleic acids from phages or plasmids [1]. The CRISPR-mediated response involves three main stages: (i) spacer acquisition (adaptation), (ii) CRISPR RNA (crRNA) expression and maturation, (iii) and interference of invading DNA. All stages were described in detail in our previous publication [2]. In the type II CRISPR/Cas9 system, Cas9 protein, driven by specificity-determining crRNA and auxiliary trans-activating RNA (tracrRNA), binds to foreign nucleic acid and cleaves both DNA strands. In 2012, Doudna and Charpentier demonstrated that Cas9 protein with crRNA and tracrRNA can generate double-strand breaks (DSB) into the target DNA sequence, enabling precise genome editing [3]. For this achievement, they were awarded the 2020 Nobel Prize in Chemistry. Although several site-specific nucleases can introduce defined alterations in the genome, CRISPR/Cas technology’s features, such as simplicity, low costs, high accuracy, and efficiency, have made it the most widely used tool for manipulating DNA. Many new types/subtypes of CRISPR systems are known, and many CRISPR/Cas-based methods and tools for genome engineering have been developed. The most important of them will be described in this manuscript.

## 2. CRISPR Systems Classification

The CRISPR systems have been categorized into two classes, six types, and 33 subtypes. The class 1 CRISPR/Cas system includes types I, III, and IV and involves multiple Cas proteins, whereas the class 2 system, which includes types II, V, and VI, only utilizes a single effector protein with multiple domains [4]. The classification of CRISPR/Cas systems is summarized in Table 1. Types I, II, and V systems recognize and cleave DNA, type VI targets RNA, and type III cuts DNA and RNA.

Type I is currently divided into seven subtypes, I-A to I-G; there are three distinct variants of the I-F subtype (I-F1, I-F2, and I-F3). The type I interference system uses the multiprotein complex termed the CRISPR-associated complex for antiviral defense (Cascade). In the type I-E system, Cascade is composed of Cse1 (Cas8), Cse2 (Cas11), Cas7, Cas5, and Cas6 proteins [4]. Recognition of target DNA by the crRNA-guided Cascade complex results in the recruitment of the Cas3 protein and degradation of the DNA.

According to the current classification, type II CRISPR/Cas systems include three subtypes, II-A to II-C; the II-C subtype has to variants (II-C1, II-C2) [4]. The type II system is based on using a single large multidomain Cas9 protein as the effector complex. Moreover, all type II CRISPR/Cas loci contain *cas1* and *cas2* genes (essential for the CRISPR adaptation) and tracrRNA (noncoding RNA, required to mature the long pre-crRNA and for interactions with Cas9 protein). The crRNA–tracrRNA–Cas9 protein complex is able to recognize and cleave the target DNA sequences. Cas9 protein contains the HNH- and RuvC-like nuclease domains, which are responsible for cleavage of the complementary and noncomplementary DNA strands, respectively. The presence of a protospacer adjacent motif (PAM), usually 2–6 bp, downstream of the target sequence is necessary for the Cas9-mediated cleavage and ensures the distinction between self and foreign DNA, thus preventing CRISPR locus targeting [5].

CRISPR/Cas type III systems are the most complex prokaryotic immune systems and utilize multi-subunit effector complexes to cleave both invading RNA and DNA. Type III systems have been classified into six subtypes: III-A to III-F [4]. These type III systems contain the gene encoding Cas10 multidomain protein with N-terminal histidine-aspartate (HD) nuclease domain (several subtypes) and two Palm domains (a form of the RNA recognition motif). The HD domain is responsible for nonspecific single-stranded DNA (ssDNA) cleavage activity, whereas the Palm domain catalyzes the conversion of ATP to cyclic oligonucleotides (cOAs) when the type III crRNA-guided effector complex recognizes the target RNA. The cOAs activate the Csm6 protein, which nonspecifically degrades RNA molecules [6].

Type IV CRISPR/Cas systems are divided into three distinct subtypes: IV-A, IV-B, and IV-C; however, their specific function is still poorly characterized [4]. Type IV lacks adaptation genes such as *cas1*, *cas2*, and *cas4*. All type IV systems contain Cas7 protein (also called Csf2) and Cas5 protein (also called Csf3), which are part of the multi-subunit complex. The subtype IV-A contains Cas6-like protein and DinG helicase, whereas subtype IV-A and subtype IV-B encode Cas8-like protein. Furthermore, subtype IV-B possesses a cysH-like gene from the family of phosphoadenosine phosphosulfate reductases, and subtype IV-C has a large subunit with a putative HD-nuclease domain [7].

Type V CRISPR/Cas systems include many subtypes (V-A to V-I, V-K, and V-U) with diverse functions [4]. V-type systems are classified based on the presence of the versatile Cas12 protein. The Cas12a ortholog (previously known as Cpf1) requires only the crRNAs to recognize the target DNA strand with PAM sequences (for the *Acidaminococcus* sp. Cas12a: TTTV, where V is A, C, or G) and generate DSBs [8,9]. Cas12a cleaves the noncomplementary and complementary strands of the targeted sequence via its active RuvC nuclease domain and produces staggered DSB. Cas12a exhibits RNase III activity, does not require a tracrRNA, and is able to generate mature crRNA [8].

Type VI CRISPR/Cas systems have been identified and divided into four subtypes: VI-A to VI-D; furthermore, there are two variants of the VI-B subtype (VI-B1, VI-B2) [4]. Type VI contains a single effector Cas13 protein (C2c2) with the two Higher Eukaryotes and Prokaryotes Nucleotide-binding (HEPN) domains. Cas13 enzyme, together with crRNA, forms an RNA-guided effector complex (tracrRNA is not required) capable of recognizing and cleaving single-stranded RNA (ssRNA) target sequences [10].

## 3. Double-Strand Break Repair

Precise genome editing is possible thanks to the introduction of DNA double-strand breaks (DSBs) into the target sequence. The two main pathways of DSB repair include nonhomologous end joining (NHEJ) and homology-directed repair (HDR). NHEJ represents the predominant repair mechanism in mammalian cells; it directly ligates DNA ends without a homologous donor template. This error-prone process often leads to the generation of small nucleotide insertions and deletions (indels) at the DSB site, which disrupts the DNA sequence and may be used for genetic knockout. In turn, HDR is a precise repair pathway that requires a donor DNA template with homology regions. The repair template, containing the desired sequence, may be provided as single-stranded oligodeoxynucleotides (ssODNs) or double-stranded DNA (dsDNA), and can introduce precise point mutations, correct a mutant gene to wild-type form, or insert genes of interest. Homology-directed repair provides accurate genome editing, but its efficiency is generally low. HDR can occur in the S and G2 phases of the cell cycle when sister chromatids are present in the cell and can be used as a repair template. The exogenous donor DNA is delivered in large amounts, so it is used more often than the sister chromatids in the repair process. HDR and NHEJ repair pathways play an essential role in CRISPR/Cas-mediated genome editing [11,12]. 

## 4. Engineered CRISPR/Cas Systems

### 4.1. CRISPR/Cas9

Among different types of CRISPR/Cas systems, the type II system (belonging to class 2) is the most widely used in genetic engineering due to its simplicity, versatility, and efficiency [2]. In the laboratory, engineered CRISPR/Cas9 technology comprises two main components: a single guide RNA (sgRNA) and Cas9 protein. The sgRNA is a short synthetic RNA created by fusing the tracrRNA and crRNA with, usually, 20 nucleotides complementary to the target sequence. The target DNA should be unique compared to the rest of the genome and should be immediately adjacent to the PAM site in the genome (for *Streptococcus pyogenes*, SpCas9: 5′-NGG-3′, where N is A, C, T, or G). The RNA-guided Cas9 enzyme, with two active HNH and RuvC nuclease domains, cleaves a target DNA sequence and generates DSB in the gene of interest [3]. Moreover, by introducing a single Cas9 protein and two or more sgRNAs, the CRISPR/Cas9 technology also allows for multiplex genome editing [13]. CRISPR multiplexing can be used for modifying multiple genes at once or deletion of large genomic regions. However, it was shown that SpCas9 can tolerate some mismatches between the guide RNA and the target DNA, resulting in off-target editing, which is still one of the significant issues in clinical applications of this system [14,15]. Therefore, Cas9 protein optimization focuses on improving the target specificity and increasing the clinical safety of Cas9. Currently, numerous high-fidelity Cas9 variants have been engineered or developed, such as SpCas9-HF1 [16], HiFiCas9 [17], evoCas9 [18], eSpCas9 [19], HypaCas9 [20], Sniper-Cas9 [21], and xCas9 3.7 [22]. However, the increased fidelity of these variants is usually associated with decreased editing efficiency [23]. It is also of importance that the number of available genome-editing sites for the most widely used *Streptococcus pyogenes* Cas9 protein is limited by the protein’s dependence on the PAM sequence (5′-NGG-3′). For this reason, additional Cas9 orthologs from various microorganisms that recognize different PAM sequences were discovered. This includes the SaCas9 protein derived from *Staphylococcus aureus* [24], CjCas9 from *Campylobacter jejuni* [25], FnCas9 from *Francisella novicida* [26], StCas9 from *Streptococcus thermophiles* [27], and NmCas9 from *Neisseria meningitides* [28]. Additionally, Cas9 variants with altered PAM compatibility were also developed, e.g., xCas9 [22], SpCas9-NG [29], SpRY [30], Cas9-EQR, and Cas9-VQR [31].

### 4.2. CRISPR/Cas12a

Another class 2 CRISPR/Cas system used in genetic engineering is type V, with the Cas12a nuclease. Unlike the Cas9 enzyme, Cas12a uses only one active RuvC catalytic domain to cleave both strands of the target dsDNA. Moreover, Cas12a recognizes T-rich PAM sequences, expanding the species and number of possible target sites compared to Cas9 with G-rich PAM. Engineered Cas12a for DNA binding and cleavage requires only the crRNA molecule with a 23 nt guide sequence [8]. As a result, the guide RNA from the CRISPR/Cas12a system is smaller (~43 nt in length) than sgRNA from CRISPR/Cas9 (~101 nt in length), which makes its chemical synthesis cheaper [32]. The activity of the Cas12a protein for pre-crRNA processing makes Cas12a ideal for multiplex genome engineering by a single transcript encoding multiple guide RNAs [33]. Furthermore, the DSB with sticky ends generated by Cas12a could provide an effective way to increase the efficiency of HDR-mediated insertion [8]. Cas12a protein seems to be more sensitive to nonseed mismatches than Cas9 and therefore is considered to induce less of an off-target effect and to be potentially safer for clinical use [34,35]. However, a limitation of this system is the low editing efficiency found in some studies [36,37].

### 4.3. CRISPR/Cas13a

The type VI CRISPR/Cas13 system is an example of an important tool for RNA editing. It was demonstrated that the Cas13a ortholog from the VI-A subtype, guided by crRNA containing a 28 nt spacer sequence, can specifically and efficiently cleave target single-stranded RNA (ssRNA) [38]. Unlike Cas9 and Cas12a, which require a PAM sequence, Cas13a recognizes the nucleotides adjacent to the target protospacer sequence at the 3′-end, known as the protospacer flanking site (PFS), and consists of a single A, U, or C. The guide RNA–target RNA duplex formation activates the catalytic site between the HEPN1 and HEPN2 domains of Cas13a protein, which subsequently cleaves the target sequence of ssRNA. Cas13a can tolerate single-nucleotide mismatches between the crRNA and target sequence, but double mismatches reduce the cleavage efficiency of the Cas13a enzyme [39].

## 5. Base Editing

Along with the development of Cas enzymes, new tools have been discovered for precise base editing without double-strand breaks or exogenous DNA templates. Base editors are constructed by fusing deaminase with Cas9 nickase (Cas9n, Cas9 with either the HNH or RuvC domain inactivated, generates single-strand breaks) or catalytically inactive “dead” Cas9 (dCas9) and allow the generation of precise point mutations in a targeted sequence through single base conversions. There are currently two widely used base editors: cytosine base editor (CBEs) and adenine base editor (ABEs).

CBEs enable the conversion of cytosines to thymines (C→T). The first-generation CBE (CBE1) complex, containing sgRNA-guided dCas9 fused to cytidine deaminase enzyme, recognizes the target DNA sequence, its dCas9 performs local denaturation of the double-stranded DNA to R-loop formation, and it exposes a short stretch of ssDNA in the noncomplementary strand for the deaminase. The cytidine deaminase removes an amino group from cytosine, converting it to uracil and causing a U–G mismatch. The DNA polymerase interprets uracil as thymine and pairs it with adenine. Ultimately, a T: A base pair is achieved [40]. The main obstacle of this system is the base excision repair (BER) cellular mechanism, which recognizes and eliminates uracil by the enzyme uracil DNA glycosylase (UDG), resulting in reversion of G: U to the original G: C pair and poor editing efficiency. To overcome this problem, the second-generation CBEs (CBE2) were developed with an additional uracil glycosylase inhibitor (UGI), which prevents the removal of the uracil base by inhibiting the action of UDG [40]. Moreover, the improvement of editing efficiency was achieved by replacing dCas9 with Cas9 nickase (Cas9n) fused to cytidine deaminase and one (third-generation CBE, CBE3) or two (fourth-generation CBE, CBE4) UGI molecules [41].

Adenine base editors work very similarly but are based on the activity of adenosine deaminase instead of the cytidine deaminase in CBEs. Because naturally occurring adenine deaminase cannot edit DNA, it was necessary to create *E. coli*-derived engineered transfer RNA adenosine deaminase (TadA) [42]. Moreover, it was demonstrated that the higher efficiency of deamination of adenine in DNA can be obtained through the use of mutated TadA (TadA*) and one wild-type enzyme (TadA–TadA* heterodimer). Finally, the ABE system consists of sgRNA-guided, catalytically impaired Cas9 nickase fused to engineered TadA–TadA* and catalyzes specific A-to-G conversions (A→G). The TadA–TadA* heterodimer deaminates adenine to inosine, which is read as G by the cell and pairs with C during DNA replication. Since G–C to A–T mutations are the most frequently reported pathogenic point mutations, ABE is especially important for therapeutics.

CBE and ABE enable 4 out of 12 possible base substitutions (purine to purine or pyrimidine to pyrimidine). Recently, C-to-G base editors (CGBE) and adenine transversion base editors (AYBE, Y = C or T base) were developed. CGBEs are created by fusing Cas9 nickase to cytidine deaminase (as in CBE) and uracil-DNA glycosylase (UNG) [43,44]. In this system, UNG removes the uracil base generated by cytosine deaminase and leaves an abasic site (apurinic/apyrimidinic site, AP site), which initiates the DNA repair process, leading to the insertion of G at the AP site. A similar strategy was also used for an adenine transversion base editor for effective A-to-T and A-to-C editing. Tong et al. constructed the AYBE by fusing an ABE with hypoxanthine excision protein N-methylpurine DNA glycosylase (MPG) [45]. MPG induces hypoxanthine group excision from the inosine generated by ABE and creates the AP site, resulting in the transversion of adenine. Further improvement of AYBE variants increased transversion editing activity to 72% for A-to-T or A-to-C editing [45].

## 6. Prime Editing

Although base editors can efficiently perform the base conversions C→T and A→G, the problem of other base conversions and small fragment insertion and deletion remains. In 2019, David Liu and coworkers described a new method of genome-editing technology, called the prime editing (PE) system, which enables all types of nucleotide conversions, targeted insertions, and deletions without an exogenous DNA template or double-strand breaks [46]. The prime editing method consists of Cas9 nickase with a reverse transcriptase (RT) derived from Moloney murine leukemia virus (M-MLV) and prime editing guide RNA (pegRNA). The pegRNA contains sgRNAs for recognizing the target sequence in the genome, primer binding site (PBS) for initiation of reverse transcription, and RNA donor sequence for the reverse transcriptase encoding the desired edit. RNA-guided Cas9 nickase introduces a single-strand nick at the noncomplementary strand of the DNA (PAM-containing strand) to expose a 3′ OH group that hybridizes with the PBS in the pegRNA, allowing the initiation of reverse transcription by the RT. The associated RT extends the 3′ flap by copying the edit sequence of the pegRNA, resulting in the formation of two intermediate, redundant ssDNA structures: the 3′ flap that contains the edited sequence and the original, unedited 5′ flap sequence. These 3’ and 5’ DNA flaps compete with each other. Although the non-edited 5′-flap is thermodynamically favored to hybridize with the complementary strand, it is degraded by cellular endonucleases, leading to the incorporation of the edited 3′ flap. The formed heteroduplex DNA, containing edited and non-edited strands, is resolved, and the desired modification is introduced into both DNA strands by ligation and DNA mismatch repair (MMR) [46].

In their publication, Anzalone et al. demonstrated three different versions of the prime editing technology: PE1, PE2, and PE3 (the first has been described above). PE2 contains engineered M-MLV RT, which increases genome-editing efficiency, whereas PE3 uses an additional sgRNA to induce a nick in the non-edited DNA strand by Cas9 nickase. Cutting the unedited strand causes the edited strand to be utilized as a template to repair a single-strand break, resulting in the permanent incorporation of the desired change on both DNA strands [46]. Although the use of the PE3 system increased the editing efficiency because both strands were cut at about the same time, an unwanted-indels problem appeared. Therefore, the PE3b version was developed that utilizes an edit-specific nicking sgRNA with a spacer that recognizes only the edited strand to reduce the indels in the non-edited DNA strand and prevents sgRNA nicking until after edited-strand-flap resolution [46]. Moreover, Chen et al. demonstrated that the inhibition of MMR enhanced the efficiency of PE and optimized the PE2 and PE3 systems by introducing a dominant negative MMR protein (MLH1dn) to generate the efficient prime editors PE4 and PE5, respectively [47].

The prime editing system was demonstrated to be more effective than CRISPR/Cas9-mediated HDR and revealed significantly fewer off-target effects than Cas9 [46]. Compared to base editing, PE offers all possible base conversions. However, prime editors cannot be used for large DNA insertions or deletions, while CRISPR/Cas9 systems can. The advantages and disadvantages of the PE, BE, and CRISPR/Cas9 systems are presented in Table 2.

## 7. Applications

The discovery of the CRISPR/Cas system has opened many avenues of research for targeting specific genes, amongst which the most important for human life seem to be human disease treatment, molecular diagnostics for infectious diseases, and food production. Clinical tests or applications of CRISPR/Cas9 systems can be categorized into two classes of therapeutic usages: ex vivo and in vivo. For ex vivo applications, a patient’s cells are isolated, manually edited, and delivered back to the same patient. The ex vivo gene-editing method has three potential clinical applications: cancer immunotherapy, treatment of hereditary diseases, and viral infection inhibition. CRISPR/Cas9-mediated editing has been used to knock out the PDCD1 gene encoding programmed cell death-1 (PD-1) as well as both the PD-1 gene and TRAC and TRBC genes encoding the endogenous T cell receptor (TCR) chains in human T cells. A phase 1 clinical trial of CRISPR/Cas9 gene-edited human T cells in non-small-cell lung cancer treatment demonstrated promising results for cancer immunotherapy [48,49]. The targeting of the B-cell Lymphoma 11A (BCL11A) gene in hematopoietic stem cells (HSCs) is suggested for patients suffering from sickle cell disease (SCD) and β-thalassemia. Gene-edited HSCs were infused into patient bodies with promising results [50]. Moreover, CRISPR/Cas9 gene-editing technology for the treatment of cystic fibrosis (CF) and Duchenne muscular dystrophy (DMD) is in the early stages of development [51,52].

Similarly, CRISPR/Cas9 could also be used to treat infectious diseases caused by microorganisms and viruses, including HIV treatment. The genome-editing system can be used in order to silence virus activity. The CRISPR/Cas9-mediated CCR5 ablation was achieved in human HSPCs and resulted in resistance to HIV-1 infection after being transplanted into mice [53]. Transplantation of CCR5-ablated HSPCs into a patient with HIV-1 infection and acute lymphoblastic leukemia resulted in complete remission for 19 months after transplantation, during which time the cells with the modified CCR5 gene persisted, and the CCR5 disruption ranged from 5.20 to 8.28% in bone marrow cells. However, the efficiency of the response was not adequate to achieve the target of a cure for HIV-1 infection [54]. Alternatively, the HIV-1 genome can be excised from the infected cells, as was shown by Yin et al. [55]. Efficient excision of the HIV-1 provirus and reduced viral RNA expression in several organs/tissues was observed in the mice model after intravenous injection. The gene construct contains the combination of sgRNAs targeting the LTRs, Gag, or Pol viral structural genes and *Staphylococcus aureus* Cas9 in the AAV vector. In 2021, Kafrelsheikh University announced a phase 1/2 trial (ClinicalTrials.gov number NCT04990557; accessed on 25 August 2023) to assess the safety of PD-1 and ACE2 knockout engineered T cells as genetically modified memory T cells capable of providing long-term immunity against COVID-19 by remembering and killing the virus if it is reintroduced. The genetically modified T lymphocytes will be selected, expanded ex vivo, and infused into patients (data not published).

In 2020, CRISPR/Cas9 was used in vivo for the first time. The components of the CRISPR/Cas9 system introduced into the genome of a virus were injected directly into the eye, near photoreceptor cells, to delete a mutation in the CEP290 gene that is responsible for Leber’s congenital amaurosis 10 (LCA10) [56]. The most important clinical applications of CRISPR/Cas9 genome editing are presented in Table 3.

The scope and capabilities of genome editing based on CRISPR/Cas9 nuclease might be expanded by using the base editing or prime editing method. In 2016, the first cytosine base editor (CBE) was used to convert C→G base pairs to T→A base pairs. Komor et al. manipulated the cellular DNA repair response to favor desired base editing outcomes, resulting in a permanent correction of ∼15–75% of total cellular DNA with minimal (typically ≤ 1%) indel formation in four transformed human and murine cell lines [40]. In 2017, adenine base editors (ABEs) were engineered and used to convert target A→T to G→C base pairs efficiently (~50% in human cells) with meager rates of indels (typically ≤ 0.1%) [42]. The base editing method enables the direct, programmable introduction of all four transition mutations without double-stranded DNA cleavage and much lower off-targets than the Cas9 nuclease-based method. According to Anzalone et al. (2019), prime editing can correct up to 89% of known genetic variants associated with human diseases. They showed that prime editing enables a variety of precise DNA edits at a wide range of positions, including all four transition point mutations, all eight transversion point mutations, insertions (up to 44 bp), deletions (up to 80 bp), and combinations of the above with a higher or similar efficiency and a much lower number of off-targets in comparison to Cas9 nuclease [46]. Recently developed base editors and prime editors enabled precise gene correction and disease rescue in multiple preclinical models of genetic disorders. Examples of therapeutic in vivo base editing and prime editing were reviewed by Newby and Liu [57].

The CRISPR/Cas12 and CRISPR/Cas13 platforms have become an essential tool in molecular diagnostics to detect specific RNA or DNA sequences derived from bacteria or viruses responsible for infectious diseases. Recombinase-mediated polymerase preamplification of DNA or RNA and subsequent Cas13- or Cas12-mediated detection via fluorescent and colorimetric readouts improved the sensitivity and specificity and reduced the time, cost, and required instruments [58,59]. In 2020, the first tool to diagnose SARS-CoV-2 was approved by the FDA (based on the SHERLOCK assay). In the same year and in 2022, two methods known as DETECTRTM and DETECTR BOOST were introduced to diagnose SARS-CoV-2 (based on the DETECTR assay). Furthermore, the CRISPR/Cas12 nuclease was used to detect human papilloma viruses (HPV) [59] and African swine fever virus (ASFV) [60].

RNA-targeting enzymes, such as Cas13, have been extensively developed for RNA-targeting applications. These enzymes might one day be used to edit disease-causing sequences of a patient’s RNAs, which could allow cells to produce healthy proteins or lower the level of a protein that is harmful due to genetic mutation. Moreover, the RNA-targeting CRISPR/Cas13 system provides an antiviral strategy against single-stranded RNA viruses. Many ssRNA viruses cause human diseases for which there are no FDA-approved therapies. The CRISPR/Cas9 nuclease can inhibit the replication of double-stranded DNA viruses or ssRNA viruses with DNA intermediates but is unable to target ssRNA viruses without DNA intermediates.

For thousands of years, all of genetics was based on selective breeding of plants and animals amongst natural genome variants. This approach has been highly successful and will continue to play a significant part in agriculture. The most crucial disadvantage of selective breeding is that chromosomes segregate randomly and there is no possibility to transfer a gene encoding a desired trait to the new variety without transferring genes encoding undesired traits. The CRISPR/Cas9 system is an exciting alternative to previously used classical breeding programs. Unlike conventional methods, CRISPR technology makes it possible to obtain specific features by introducing DNA material that occurs naturally in a given crop family without the introduction of DNA material bearing unwanted genetic information. The use of CRISPR/Cas9 in the food sector will bring many benefits to agriculture because it is a cheaper, faster, more straightforward, and more precise method than selective breeding. Thanks to the use of CRISPR/Cas9 it was possible, among other examples, to create a variety of corn with lower water requirements [61], to increase the vitamin D content in tomatoes [62], to breed cattle for meat production with extremely slick, short hair, which is said to help the animals cope with hot weather more effectively [63], and to breed MSTN-edited Hu sheep with the double-muscled phenotype [64].

## 8. Challenges and Future Perspectives

While choosing an approach to achieve the assumed scientific or application goal, we are guided mainly by the advantages and effectiveness of the method. However, the system’s disadvantages and weak points must also be considered. As in the case of other genome-editing methods, we can also indicate several limitations and problems to overcome for CRISPR systems. These challenges become extremely important in the context of CRISPR technology’s therapeutic and clinical applications. The risks associated with the exploitation of the CRISPR/Cas9 system are primarily associated with methodological challenges and nontechnological aspects. Methodological challenges refer to problems strictly related to technology, whereas nontechnological aspects embrace ethical issues. In this paper, we focus and shed light on some challenges.

The Cas9 proteins used in the CRISPR system originate from infectious disease agents in humans, for example, *Streptococcus pyogenes* (SpCas9) and *Staphylococcus aureus* (SaCas9) [65]. These two are the most often used Cas9 proteins in practice due to the ease of packaging them into vectors. Cas9 protein is not the only factor posing a risk of immunological reaction against the CRISPR system. The sgRNAs can also trigger an innate immune response, but chemically modified 5′-ends help to avoid recognition effectively [66]. Delivery systems based on viral vectors such as adeno-associated viruses (AAV) are a third severe threat. Preexisting and inducible adaptive immune responses associated with viral vectors are widely described problems in gene therapy and vaccinology; thus, we decided to exclude this issue in our paper [67].

It looks a bit like playing with fire when immune-activating agents are engaged to evoke a therapeutic effect. The unintended ambiguous activity of Cas9 protein creates an immunological challenge to be overcome. Cas9 proteins are connected to both preexisting humoral and cell-mediated adaptive immune responses. This preexisting adaptive immune response can be triggered by similarities to Cas9 proteins originating from different bacteria and by similarities to nonrelated proteins [68]. One of the strategies to conquer problems connected with an immune system activation is to treat defects (if possible) with the CRISPR platform before the patient is immunized with infectious agents and starts to produce antibodies against Cas9 protein. It narrows down the application of the CRISPR system to diseases, including thalassemia and sickle cell anemia, detectable in fetal life or early childhood [69]. If other diseases, e.g., neuroblastoma, are diagnosed later in life, the immunological safety of the CRISPR system depends on whether the human body has been immunized before assessment [70]. In the case of individuals with preexisting immunity for repeated treatment, the search for a rarely occurring donor of Cas9 proteins can be a solution. The above-mentioned solutions are not technologically astonishing, but they may turn out to be effective, assuming high efficiency and earliness of diagnostics. It is also worth thinking about harnessing in silico tools to predict the level of immunogenicity based on binding-affinity algorithms [71]. As scientists do not want CRISPR systems to suffer the same initial collapse as gene therapy did, some protective solutions to mitigate immunogenicity are being adopted from that system, for example, the transfer of Treg cells or immunogenic epitopes masking [68]. The very safe approach, if applicable (not for systemic delivery), is to edit genes ex vivo and then transfer them as treatment into organisms [72]. For instance, in 2019, Rio et al. reported successful engraftment of gene-corrected hematopoietic stem cells in nonconditioned patients with Fanconi anemia [73]. Targeting the site of CRISPR system components delivery is also a promising approach. So-called immune-privileged organs in the human body such as the eyes, brain, testicles, and placenta as well as the fetus are the sites with reduced risk of graft rejection. This means that some disorders connected to these particular regions can be managed with a CRISPR system with lower immunological threat. As eyes are immune-privileged sites, there are plenty of reported applications of CRISPR technology in ophthalmological disorder treatment, as in model animals (corneal dystrophy, glaucoma, congenital cataract, Leber’s congenital amaurosis, retinitis pigmentosa, Usher syndrome, fundus neovascular disease, proliferative vitreoretinopathy, retinoblastoma, and other eye diseases) [74]. Despite an immature immune system and being immune-privileged, the fetus is still very challenging because of the high off-targeting rate and possible loss of DNA fragments [75]. Other strategies being harnessed to overcome the immunogenicity of CRISPR components in systemic delivery include the coadministration of immunosuppressive drugs or the administration in immunocompromised/immunodeficient individuals and also controlling the activity duration of the CRISPR system in organisms, e.g., by transient expression [68].

The immunogenicity of the above-mentioned components is not the only problem to be dealt with while using the CRISPR system. Off-targeting has been the Achilles’ heel since the CRISPR system was developed. Off-targets are side effects produced by an unwitting cleavage activity of Cas9 protein in nontargeted sequences. The unintentional cleavages in similar target sequences are generated because Cas9 accepts up to three sgRNA/gDNA mismatches [76]. According to the region covered, there are two types of mismatches: proximal, connected with impaired substrate DNA binding and R-loop formation, and distal, connected with the production of a catalytically nonfunctional complex [77]. The sgRNA-independent factors have been also been proved to cause off-targets; thus, they must also be managed. Consequences of off-targets can vary in effect according to the significance of unwittingly cut sequences leading to lethal effect, loss of gene function, or even carcinogenesis induction [78]. The steps that can be undertaken by scientists to avoid off-targeting include bioinformatics attempts (gRNA designing software, off-targets predicting software) and/or the engagement of other biochemical components (Cas9 nickases, anti-CRISPR proteins—Acr-p) [70]. For instance, well-designed gRNA can reduce the number of off-targets 5000-fold without losing its efficiency [79]. gRNAs should not be longer than 20 bp (a significant rise in off-targets) and not shorter than 17 bp (a substantial loss of specificity). There are two categories of methods for detecting off-targets: biased and unbiased. Biased methods are connected with in silico modeling and computational detection of off-target sites. The obtained in silico data analysis checked and validated by in vitro/in vivo methods and platforms helps to improve software algorithms, which makes these tools more and more sophisticated and accurate. The algorithms are based on the knowledge obtained through observations, but not everything can be predicted; thus, unbiased detection of off-target sites should also be applied. Unbiased off-target analysis can be performed using different strategies such as cross-linking chromatin immunoprecipitation of endonuclease-mutant Cas9 (ChIP-dCas9), integrative-deficient lentiviral vectors (IDLV) capture, detection of off-target cleavage by CRISPR/Cas9 using GUIDE-seq methods, LAM-PCR-based high-throughput genomewide translocation sequencing (LAM-HTGTS), or whole genome sequencing (WGS) [80]. As mentioned above, other biochemical components can be used to reduce levels of off-targeting. Cas9 nickases, mutated variants of Cas9, show higher efficiency in on-targets and lower effect in off-targets. Cas9n requires two sgRNAs to act [81]. Viruses produce anti-CRISPR proteins (Acr-p) to deactivate prokaryotic defense systems based on CRISPR systems. Anti-CRISPR-associated proteins (Aca-p) act antagonistically and inhibit the transcription of anti-CRISPR proteins. The above-mentioned proteins can thus modulate the lifespan of the CRISPR system in organisms to reduce off-targeting [82].

Future perspectives for the CRISPR system are deeply associated with society’s acceptance or refusal of this powerful tool. On the other hand, the scientific applications of the CRISPR platform are society-independent, and there is no doubt that they will be continued no matter what. Ethical issues concerning the CRISPR system are generally related to all techniques that are able to alter genetic information. Those ethical problems usually result from real or unjustifiable risks connected with new technologies. Other aspects are the religious and cultural ones. When it comes to humans, certain threats have been concluded on the basis of in vitro and in vivo animal experiments, generating certain ethical issues. Side effects, including off-targeting, immunological reactions, and genetic mosaicism, and financial costs generate the following concerns: safety, accessibility, eugenics, regulations, legislation, and patenting [83]. Some of these concerns are convergent with those connected to gene therapy (i.e., safety and accessibility). In clinical applications, the nonheritable therapeutic effect of the CRISPR system on somatic cells is much easier to accept by society than human germ cell genome editing. Before any possible CRISPR therapeutic applications, one should consider all of the technology’s pros and cons and then be allowed to roll the dice individually. Intense public backlash against germline modifications is linked to its potential to encourage the reproduction of humans with desirable traits, which can lead to eugenics sooner or later. In this situation, the most reasonable solution for reconciling the interests of individuals and society is to establish wise and humane laws. Currently, local and international laws do not keep pace with rapidly developing technologies, and this state of the art provokes community discord.

## Figures and Tables

**Table 1:** Characteristics of different types of CRISPR/Cas systems.

Class	Type	Subtypes	Effector Complex	tracrRNA	Signature Protein	Target Substrate
1	I	A, B, C, D, E, F, G	multiple subunits	no	Cas3	DNA
	III	A, B, C, D, E, F,	multiple subunits	no	Cas10	DNA/RNA
	IV	A, B, C	multiple subunits	no	unknown	unknown
2	II	A, B, C	single unit	yes	Cas9	DNA
	V	A, B, C, D, E, F, G, H, I, K, U	single unit	yes ^1^	Cas12	DNA/RNA ^2^
	VI	A, B, C, D	single unit	no	Cas13	RNA

^1^ For subtypes: B, E, F, G, K. ^2^ For subtype V-G.

**Table 2:** The comparison of the CRISPR/Cas9 system, base editor, and prime editor.

	CRISPR/Cas9 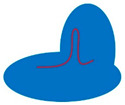	Base Editor 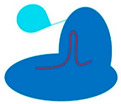	Prime Editor 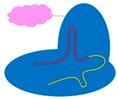
Components	Cas9 nuclease sgRNA Donor DNA (for HDR)	Fusion of dCas9 or Cas9n and deaminase sgRNA	Fusion of Cas9n and reverse transcriptase pegRNA
DNA breaks	DSB	SSB (for Cas9n)	SSB
Possible modifications	All precise modifications Large DNA insertions or deletions	Transition mutations (C→T, G→A, A→G, and T→C)	All precise modifications
Advantages	High cleavage efficiency of Cas9 Possibility of introducing large DNA insertions (transgenes) or large deletions	Fewer indel byproducts than CRISPR/Cas9 or prime editors High editing efficiency	More targeting flexibility than base editors (all types of base conversion and small deletions and insertions) High editing precision
Potential obstacles	Low-efficiency homologous recombination processes Off-target cleavage	Transversion, insertion, and deletion are not possible May induce off-target mutations in both DNA and RNA Bystander editing	Potential transcriptomic dysregulation Relatively low editing efficiency

**Table 3:** Examples of CRISPR/Cas9 application in ex vivo and in vivo clinical trials.

Disease Entity	Class of Therapeutic Usage	Application	Gene Construct Delivery Method/Phase of Clinical Trial	Targeted Gene/Cell Type	References
Metastatic non-small-cell lung cancer	Ex vivo	Cancer immunotherapy	Electroporation/phase 1 (ClinicalTrials.gov number NCT03399448)	Knockout of the *PD-1* gene or *TRAC*, *TRBC*, and *PD-1* genes in T cells	[48,49]
Sickle cell anemia β-thalassemia	Ex vivo	Hereditary disease	Electroporation/phase 2/3 (ClinicalTrials.gov number NCT03655678)	Knockout of the B-cell Lymphoma 11A (BCL11A) gene in hematopoietic stem/progenitor cells (HSPCs)	[50]
AIDS	Ex vivo	Viral replication inhibition	HSPC transplantation/not applicable (ClinicalTrials.gov number NCT03164135)	Knockout of the *CCR5* gene in HSPCs	[53,54]
AIDS	In vivo	HIV-1 proviral DNA excision	Intravenous injection/mice model	Excision of the HIV-1 provirus and reduced viral RNA expression in several organs/tissues in mice model	[55]
COVID-19 respiratory infection	Ex vivo	Induction of long-term immunity against COVID-19	Phase 1/2 (ClinicalTrials.Gov number NTC04990557)	*PD-1* and *ACE2* knockout T cells	Not published
Leber’s congenital amaurosis 10	In vivo	Hereditary disease into the subretinal space	Direct injection	*CEP290* gene	[56]

## Data Availability

Not applicable.
